# Association between atorvastatin and erectile dysfunction: a comprehensive analysis incorporating real-world pharmacovigilance and Mendelian randomization

**DOI:** 10.3389/fphar.2024.1382924

**Published:** 2024-04-29

**Authors:** Kaiqin Chen, Hesen Huang, Yongtai Chen, Weizhen He

**Affiliations:** ^1^ Department of Neurosurgery, Xiang’an Hospital of Xiamen University, Xia Men, Fu Jian, China; ^2^ Department of Otolaryngology-Head and Neck Surgery, Xiang’an Hospital of Xiamen University, Xia Men, Fu Jian, China; ^3^ Department of Hepatobiliary Surgery, The Affiliated Longyan First Hospital of Fujian Medical University, Longyan, Fujian, China

**Keywords:** atorvastatin, erectile dysfunction, real-world data analysis, adverse drug reaction, Mendelian randomization

## Abstract

**Background:**

Atorvastatin is a commonly prescribed medication for the prevention of cardiovascular diseases. Recent observational studies have suggested a potential association between atorvastatin use and the occurrence of Erectile Dysfunction (ED). In this study, we aimed to explore the relationship between atorvastatin and ED using real-world data from the FAERS database and employed Mendelian randomization to assess causality.

**Methods:**

To evaluate the disproportionality of atorvastatin in relation to ED, we conducted several pharmacovigilance analyses, including odds ratio (ROR), proportional reporting ratio (PRR), Bayesian Confidence propagation neural network (BCPNN), and gamma-Poisson contractile apparatus (GPS). Additionally, we employed Mendelian randomization to investigate the causal relationship between atorvastatin and ED.

**Results:**

Pharmacovigilance disproportionality analysis revealed a significant association between atorvastatin and ED, as indicated by the following results: ROR [3.707078559, 95% CI (3.33250349, 4.123756054)], PRR [3.702969038, χ2 (669.2853829)], IC [1.870490139, IC025 (1.702813857)], and EBGM [3.656567867, EBGM05 (3.28709656)]. Furthermore, the two-sample Mendelian randomization analysis provided evidence supporting a causal relationship between atorvastatin use and ED, with an inverse variance weighted estimate of β = 3.17 (OR = 23.91, *p* = 0.02 < 0.05).

**Conclusion:**

Based on comprehensive analyses incorporating pharmacovigilance and Mendelian randomization, our findings suggest that atorvastatin use is associated with an increased risk of ED and indicate a causal relationship. These results emphasize the importance of considering potential adverse effects, such as ED, when prescribing atorvastatin for cardiovascular disease prevention. Further research and clinical monitoring are warranted to better understand the underlying mechanisms and develop appropriate strategies to mitigate this side effect.

## 1 Introduction

Atorvastatin, through 3-hydroxy-3-methylglutaryl coenzyme A (HMG-CoA) reductase inhibition, not only lower LDL-C levels but also demonstrate pleiotropic effects like anti-inflammatory activity, endothelial function improvement, and reduction of atherosclerosis, essential in treating atherosclerotic cardiovascular diseases (ASCVDs) by interrupting inflammation within plaques and suppressing inflammatory mediator secretion (Takata et al., 2016). Consequently, it effectively lowers lipid levels within the body (Winkler et al., 2004). Despite the widespread use of statins for their efficacy in managing cholesterol production, there are lingering concerns regarding their potential side effects. A study involving eight patients prescribed various statins (simvastatin, fluvastatin, pravastatin, and atorvastatin) revealed a decrease in libido during treatment. Further examination of 2 patients assessed the patients’ sex hormone levels, showing a significant reduction in testosterone levels (de Graaf et al., 2004). Zekeriya’s study on the effects of rosuvastatin and atorvastatin on erectile dysfunction in hypercholesterolemic patients found that rosuvastatin had no impact on erectile dysfunction, while atorvastatin was shown to worsen erectile dysfunction (Nurkalem et al., 2014). Therefore, the effect of statins on sexual function raises concerns.

Apart from reducing lipids, atorvastatin has been observed to hinder the growth and movement of vascular smooth muscle cells, while also encouraging apoptosis. Furthermore, it improves blood rheology and viscosity, thereby enhancing cardiac, vascular endothelial, and coagulation functions (Beltrán Romero et al., 2021). Due to the fact that elevated levels of low-density lipoprotein cholesterol (LDL-C) pose an independent risk for cardiovascular disease, decreasing LDL-C levels has been connected with a lower likelihood of experiencing significant cardiovascular events (Gencer et al., 2020). Multiple research investigations have shown that aggressive methods to lower lipid levels can markedly diminish the expected risk of atherosclerotic cardiovascular disease over a span of 30 years (Pencina et al., 2020).

The European Society of Cardiology/European Atherosclerosis Society guidelines suggest that despite already low LDL-C levels, reducing LDL-C can effectively decrease cardiovascular disease (CVD) risk. The extent of risk reduction in CVD is directly linked to the extent of LDL-C level changes. The actual benefits of lowering LDL-C depend on the individual’s ASCVD risk profile and the absolute reduction in LDL-C levels. Therefore, even a slight decrease in LDL-C could be beneficial for individuals at high or very high cardiovascular risk (Visseren et al., 2022). Accordingly, atorvastatin assumes a crucial role in the prevention and management of cardiovascular and cerebrovascular diseases, emerging as a cornerstone in therapeutic strategies for these clinical contexts.

Erectile dysfunction (ED) is a common issue impacting considerable portion of men globally, leading to significant distress and affecting their general wellbeing. It is defined by the persistent challenge in attaining or sustaining an erection suitable for satisfactory sexual activity. The development of ED is influenced by a range of factors, encompassing physiological, psychological, and lifestyle-related elements (Salonia et al., 2021).

However, concerns have arisen regarding the potential impact of atorvastatin on sexual function, particularly its association with ED. Some clinical observations suggested a possible link between atorvastatin and the development or exacerbation of ED symptoms (Rizvi et al., 2002; Do et al., 2009). These reports have raised questions about the mechanisms through which atorvastatin may affect erectile function, prompting further investigation into this potential relationship.

Understanding the potential relationship between atorvastatin and ED is of great clinical importance. As atorvastatin is widely prescribed and often used long-term, it is crucial to assess its impact on sexual function to ensure comprehensive patient care. Furthermore, identifying any association between atorvastatin and ED can guide healthcare professionals in managing patients who experience sexual dysfunction while on this medication.

Adverse events (AE) frequently occur with drug use, although they cannot always be directly attributed to the drug. However, a large-scale statistical, biological, and clinical analysis of AEs may reveal related causes and effects, known as ADRs (Shu et al., 2022). To facilitate this, the United States established the FDA Adverse Event Reporting System (FAERS) database in 2012. The FAERS database documents a large number of AEs and medication errors associated with human drugs and therapeutic biologics. Such research can explore ADRs and provide evidence for subsequent safe drug use.

Due to the nature of ADRs, causal reasoning is challenging. However, Nevertheless, Mendelian randomization (MR) analysis offers a way to address the constraints of conventional observational studies. MR utilizes information on genetic variations to infer causation between exposure and outcome, assessing whether an observational association is consistent with a causal effect (Walker et al., 2017). It is based on the principles of random gamete division and genetic variation, simulating the random assignment of research subjects (Levin and Burgess, 2024). Due to the challenges in determining an appropriate experimental methodology, there is a lack of studies investigating a direct causal relationship between atorvastatin and ED. Therefore, this study combines the advantages of pharmacovigilance analysis and MR analysis to explore the relationship between atorvastatin and ED.

## 2 Materials and methods

### 2.1 Data sources

Pharmacovigilance disproportional analysis data was pulled from the openly accessible FAERS database. The data extracted in this study covered all data in ASCII packets from Q1 2004–Q1 2023 (77 quarters), which were cleaned and analysed using SAS software version 9.4 (Yavne et al., 2023).

Treatment/medication code: atorvastatin data was pulled from The UK Biobank database. The dataset contained ncase: 13,851; ncontrol: 449,082, and was collected in 2018 (Sudlow et al., 2015). Erectile dysfunction data was also pulled from the EBI database (http://www.ebi.ac.uk/). The dataset contained ncase:6,175; ncontrol: 217,630, collected in 2018 (Bovijn et al., 2019). LDL-C data was pulled from the development GAWS IEU database (https://gwas.mrcieu.ac.uk/). The sample size was 173,082, and the data had been collected since 2013 (Dataset: ieu-a-300) (Willer et al., 2013).

### 2.2 Data processing

According to the FDA’s recommended method for removing duplicate reports, select the PRIMARY-ID, CASE-ID, and FDA_DT fields from the DEMO table. Sort the data based on CASE-ID, FDA_DT, and PRIMARY-ID. For reports with the same CASE-ID, keep the one with the highest FDA_DT value. If the CASE-ID and FDA_DT are the same, keep the one with the highest PRIMARY-ID value.

Since the first quarter of 2019, each quarterly data package contains a list of reports to be removed. After deduplicating the data, remove the reports based on the CASEID in the deletion report list.

In the FAERS database, adverse reaction names are recorded using the Preferred Term (PT) terminology from the Medical Dictionary for Regulatory Activities (MedDRA). MedDRA dictionary is updated in March and September each year, which may involve adjustments in the PT hierarchy and changes in the System Organ Class (SOC). Therefore, it is necessary to use the latest version of the MedDRA dictionary to correct the PT names in the FAERS database and obtain the updated SOC and PT from the latest version of the MedDRA dictionary.

### 2.3 Data analysis

#### 2.3.1 Pharmacovigilance disproportionality analysis

Pharmacovigilance disproportionality analysis was conducted to explore the potential association between atorvastatin and ADRs using descriptive statistics. In our investigative study, we utilized this approach to identify any signals of disproportionality between atorvastatin and ADR. Four primary methods, namely, reporting odds ratio (ROR), proportional reporting ratio (PRR), Bayesian confidence propagation neural network (BCPNN), and multi-item gamma Poisson shrinker (MGPS), were employed to assess the relationship between atorvastatin and ADR (Bate et al., 1998; van Puijenbroek et al., 2002; Rothman et al., 2004; Shu et al., 2022). These methods are commonly used in pharmacovigilance studies to evaluate the potential association between a drug and AEs.This method is further detailed below.

Ⅰ. ROR method:

$$\text{ROR}=\frac{\left(a/c\right)}{\left(b/d\right)}=\frac{ad}{bc}$$

$$\text{SE}\left(\text{lnROR}\right)=\sqrt{\;\left(\frac{1}{a}+\frac{1}{b}+\frac{1}{c}+\frac{1}{d}\right)\;}$$

$$95\%\text{CI}=e^{\ln\left(\text{ROR}\right)\pm 1.96\sqrt{\;\left(\frac{1}{a}+\frac{1}{b}+\frac{1}{c}+\frac{1}{d}\right)\;}}$$

The criteria for positive signals: a≥3, 95% confidence interval (CI; the lower limit) > 1

Ⅱ. PRR method:

$$\text{PRR}=\frac{a/\left(a+b\right)}{c/\left(c+d\right)}$$

$$\chi 2=\frac{\;{\left(\left|\text{ad}-\text{bc}\right|-\frac{a+b+c+d}{2}\right)}^{2}\left(a+b+c+d\right)}{\left(\;a+b\right)\left(a+c\right)\left(c+d\right)\left(b+d\right)}$$

Ⅲ. BCPNN method:

$${IC=\log}_{2}\frac{p\left(x,y\right)}{p\left(x\right)p\left(y\right)}=\log_{2}\frac{a\left(a+b+c+d\right)}{\left(a+b\right)\left(a+c\right)}$$

$${E\left(IC\right)=\log}_{2}\frac{\left(a+\gamma 11\right)\left(a+b+c+d+\alpha \right)\left(a+b+c+d+\beta \right)}{\left(a+b+c+d+\gamma \right)\left(a+b+\alpha 1\right)\left(a+c+\beta 1\right)}$$

$$V\left(\text{IC}\right)=\frac{1}{{\left(\ln2\right)}^{2}}\left\{\left[\frac{\left(a+b+c+d\right)-a+\gamma -\gamma 11}{\left(a+\gamma 11\right)\left(1+a+b+c+d+\gamma \right)}\right]+\left[\frac{\left(a+b+c+d\right)-\left(a+b\right)+\alpha -\alpha 1}{\left(a+b+\alpha 1\right)\left(1+a+b+c+d+\alpha \right)}\right]+\left[\frac{\left(a+b+c+d\right)-\left(a+c\right)+\beta -\beta 1}{\left(a+c+\beta 1\right)\left(1+a+b+c+d+\beta \right)}\right]\right\}$$

$$\gamma =\gamma 11\frac{\left(a+b+c+d+\alpha \right)\left(a+b+c+d+\beta \right)}{\left(a+b+\alpha 1\right)\left(a+c+\beta 1\right)}$$

$$IC–2SD=E\left(IC\right)–2\sqrt{V\left(IC\right)}$$

α1=β1=1;α=β=2;γ11=1

The criteria for positive signals:

1) (−): IC–2SD ≤ 0;
2) (+):0<IC–2SD ≤ 1.5;
3) (++): 1.5<IC–2SD ≤ 3;
4) (+++):IC–2SD > 3.
Ⅳ. MGPS method:

$$\text{EBGM}=\frac{a\left(a+b+c+d\right)}{\left(a+c\right)\left(a+b\right)}$$

$$95\%\text{CI}=e^{\ln\left(\text{EBGM}\right)\pm 1.96\sqrt{\;\left(\frac{1}{a}+\frac{1}{b}+\frac{1}{c}+\frac{1}{d}\right)\;}}$$

Criteria for positive signals: EBGM05 > 2.

The meanings of a,b,c and d can be seen in Table 1.

**TABLE 1:** 2 x 2 matrix.

	Target ADR	Non-target ADR	Total
Atorvastatin	a	b	a+b
Non-atorvastatin	c	d	c+d
Total	a+c	b+d	n=a+b+c+d

1) a, Frequency of target adverse reactions for the target drug population.

2) b, Total adverse reactions occurred in the target drug population.

3) c, Total number of target adverse reactions.

4) d, Total number of adverse reactions occurring in the background population.

#### 2.3.2 Mendelian randomisation study

According to MR theory, Instrumental variables (IVs) need to meet the following three assumptions:

The IVs must be strongly correlated with the exposure (correlation hypothesis).

The IVs should be independent of confounding factors that affect the exposure-outcome relationship (independence hypothesis).

The IVs can only influence the occurrence of the outcome through the exposure factors and not through any other means (exclusivity hypothesis).

For the MR data analysis, we utilized the “TwoSampleMR” package in the R. The inverse-weighted variance analysis was used to determine the causal relationship between the exposure factors and the outcome (Chen et al., 2020; Zagkos et al., 2022; Wang et al., 2023). To assess heterogeneity among the study samples, MR-Egger and inverse variance-weighted functions in Cochran’s Q test were utilized, with a significance level of *p* >0.05 indicating non-heterogeneity. The pleiotropy was analyzed using the “mr_pleiotropy_test” function from the R package of the same name, with a significance level of *p* >0.05 indicatingabsence of horizontal pleiotropy.

## 3 Results

### 3.1 Descriptive results of pharmacovigilance analysis

The signal detection analysis of atorvastatin at the SOC level is presented in Table 2, revealing a comprehensive overview of its AEs. The results demonstrate that atorvastatin-related AEs are widespread, affecting a total of 27 organ systems, thus indicating their relatively common occurrence. Notably, the highest number of reported AEs were Musculoskeletal and Connective Tissue Disorders (n = 38,478), followed by General Disorders and Administration Site Conditions (n = 29,960), Investigations (n = 21,934), Nervous System Disorders (n = 20,761), and Metabolism and Nutrition Disorders (n = 15,099). These findings are consistent with the clinical observations encountered in our practice. Additionally, within the categories of reproductive system and breast disorders, we identified five distinct positive signals of adverse reactions, with a total of 1,213 reported cases.

**TABLE 2:** The PT signal of atorvastatin under Systematic Organ classification (SOC).

SYSTEM ORGAN CLASS(SOC)	PT (n)	Case report (n)	Case proportion (%)
Musculoskeletal and connective tissue disorders	52	38478	16.93
General disorders and administration site conditions	12	29960	13.18
Investigations	92	21934	9.65
Nervous system disorders	37	20761	9.14
Metabolism and nutrition disorders	10	15099	6.64
Gastrointestinal disorders	12	15053	6.62
Skin and subcutaneous tissue disorders	22	9306	4.09
Injury, poisoning and procedural complications	21	8961	3.94
Psychiatric disorders	3	8528	3.75
Respiratory, thoracic and mediastinal disorders	11	8159	3.59
Cardiac disorders	25	7906	3.48
Hepatobiliary disorders	33	6766	2.98
Renal and urinary disorders	7	5582	2.46
Immune system disorders	4	4944	2.18
Infections and infestations	13	4758	2.09
Vascular disorders	15	4671	2.06
Eye disorders	9	3792	1.67
Neoplasms benign, malignant and unspecified (incl cysts and polyps)	11	2298	1.01
Blood and lymphatic system disorders	5	2175	0.96
Surgical and medical procedures	13	1916	0.84
Ear and labyrinth disorders	4	1601	0.70
Product issues	5	1266	0.56
Reproductive system and breast disorders	5	1213	0.53
Social circumstances	5	1179	0.52
Endocrine disorders	0	477	0.21
Congenital, familial and genetic disorders	12	306	0.13
Pregnancy, puerperium and perinatal conditions	0	174	0.08
	438	227263	100.00

The analysis identified several positive signals of adverse reactions, namely, Erectile Dysfunction, Genital Swelling, Haematospermia, Nipple Swelling, and Pelvic Floor Muscle Weakness. Among these, Erectile Dysfunction exhibited notable statistical indicators: (ROR:3.707078559,95%CI(3.33250349–4.123756054), (PRR:3.702969038,χ2 (669.2853829)), (IC:1.870490139,IC025(1.702813857), (EBGM:3.656567867,EBGM05 (3.28709656)) All four types of Erectile Dysfunction tested positive, further affirming its significance as an ADR. Therefore, Erectile Dysfunction is considered a valuable ADR to be taken into account (Table 3).

**TABLE 3:** The PT signal of Atorvastatin under Reproductive system and Breast Disorders

PT	ROR (95%CI)	PRR (95%C)	χ^2^	IC (95%CI)	IC Signal strength	EBGM (EBGM05)
Erectile dysfunction	3.707078559 (3.33250349–4.123756054)	3.702969038 (3.329340793–4.118526923)	669.2853829	1.870490139 (1.702813857–2.015942789)	(++)	3.656567867 (3.28709656)
Genital swelling	5.579288982 (3.216627557–9.677360834)	5.579027035 (3.216575735–9.676608053)	47.60524887	2.449327906 (1.266489183–2.82994912)	(+)	5.461616085 (3.1487856)
Haematospermia	4.034806581 (2.221949702–7.326747372)	4.03465969 (2.221932067–7.326272055)	24.63902939	1.99203792 (0.829036636–2.511816228)	(+)	3.97798523 (2.190658442)
Nipple swelling	8.60952786 (4.245936854–17.4576242)	8.609259993 (4.245906761–17.45666163)	51.70388947	3.055250025 (1.211234572–3.176435841)	(+)	8.312313262 (4.099360359)
Pelvic floor muscle weakness	13.56863618 (6.638947561–27.73148699)	13.56819375 (6.638888824–27.72992386)	87.53328957	3.679447689 (1.472259075–3.459437489)	(+)	12.81221217 (6.268839667)

A demographic analysis of the patient population experiencing erectile dysfunction revealed that the largest proportion of participants (34.49%) belonged to the middle-aged group (45–65 years), with a total of 119 individuals. The average age of the participants was 58.39 years. Consumer reports accounted for the highest number of cases, with 124 individuals (35.94%), followed by doctors, with 107 individuals (31.01%).

Furthermore, the analysis identified the top five reporting countries for these cases, which were the United States, United Kingdom, Netherlands, Spain, and Germany (Table 4).

**TABLE 4:** The features of reports associated with Atorvastatin-ED.

	Case report (N)	Case proportion (%)
**Age**		
< 18	0	0.00
≥18, <45	27	7.83
≥45, <65	119	34.49
≥65, <75	49	14.20
75≤	22	6.38
Not Specified	128	37.10
**Age (quantitative)**		
Mean (SD)	58.39	
Median (Q1,Q3)	58.00 (50.00, 67.00)	
Min, Max	28.00, 82.00	
**Reporter**		
Consumer	124	35.94
Lawyer	2	0.58
Not Specified	27	7.83
Other health-professional	46	13.33
Pharmacist	39	11.30
Physician	107	31.01
**Country (TOP5)**		
United States of America(%)	106	30.72
United Kiongdom(%)	70	20.29
Netherlands(%)	21	6.09
Spain(%)	17	4.93
Germany(%)	13	3.77
**Outcome**		
OT	227	65.80
NOT SPECIFIED	50	14.49
DS	22	6.38
HO;OT	16	4.64
DS;OT	9	2.61
HO	4	1.16
**Serious report**		
Serious	295	85.51
Non-Serious	50	14.49

**FIGURE 1:**
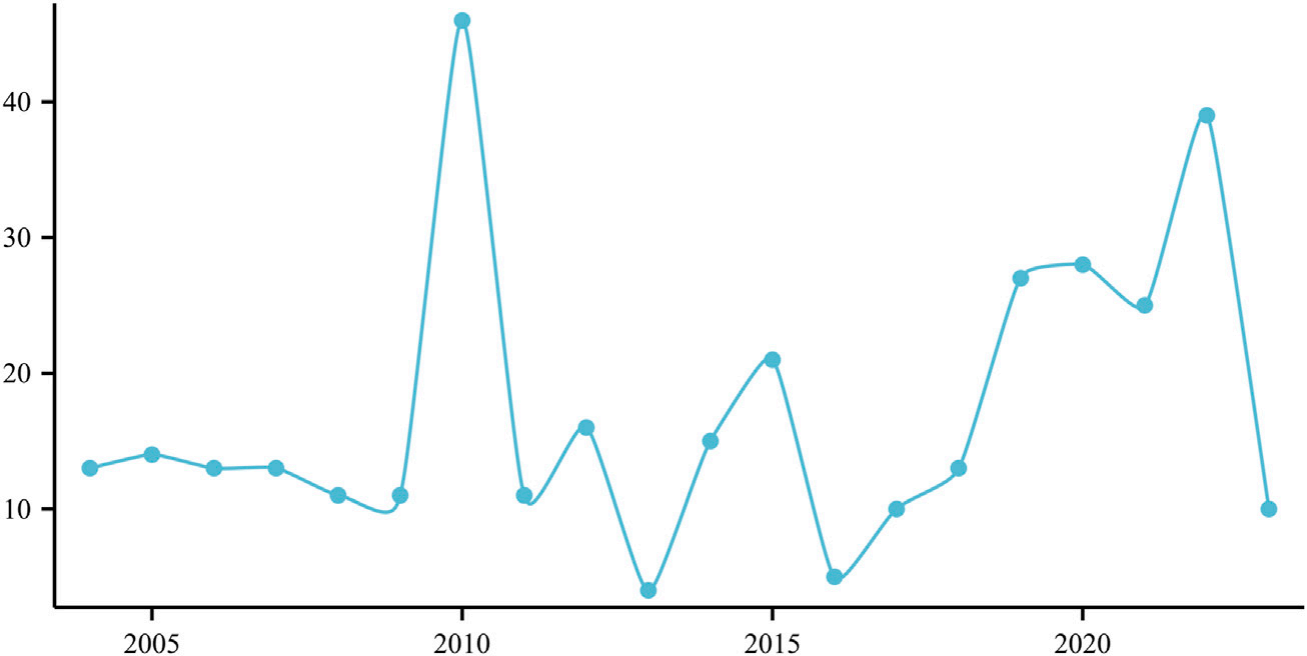
The broken line of the reporting year (Atorvastatin-ED).

The frequency of annual reports documenting ED associated with the use of atorvastatin is depicted in Figure 1, revealing a notable peak of 49 cases reported in 2010 (Figure 1).

**FIGURE 2:**
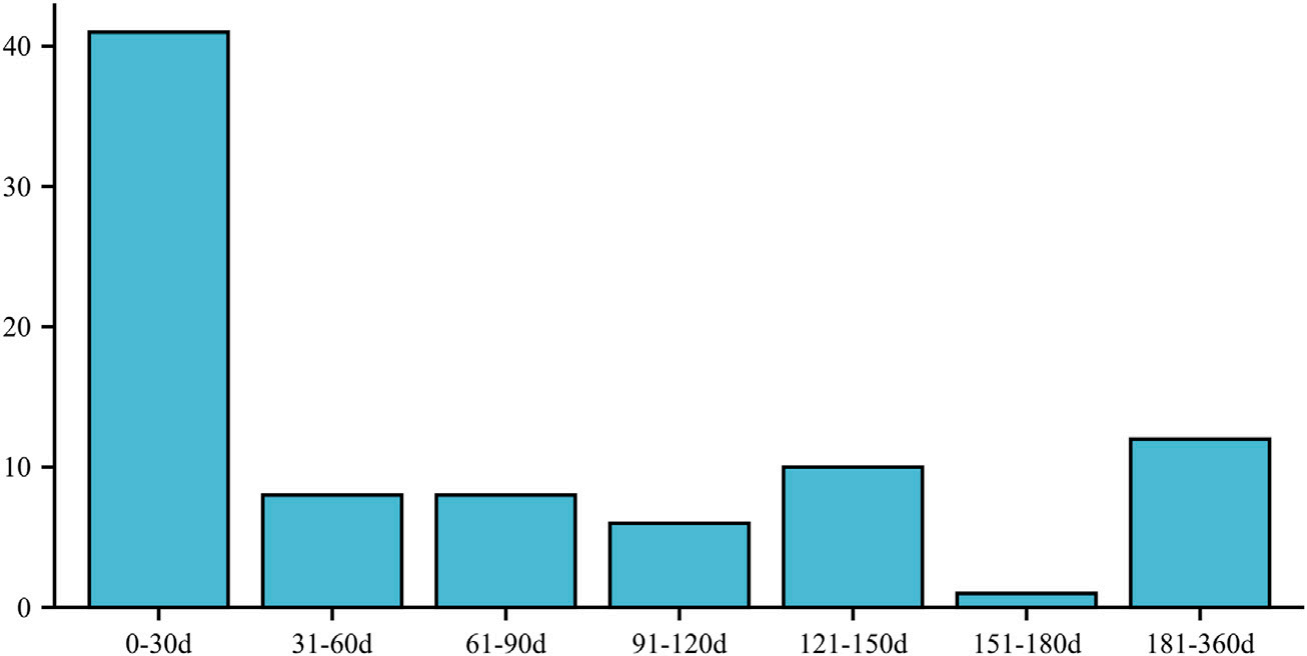
ED onset time - medication date (days).

Furthermore, the onset of ED in individuals taking atorvastatin was most commonly observed within the first 30 days (n = 41) and between 181 and 360 days (n = 12) after initiating the medication. These time intervals indicate the periods during which ED symptoms were most likely to manifest following atorvastatin use (Figure 2).

### 3.2 Results of mendelian randomisation analysis

#### 3.2.1 Atorvastatin-erectile dysfunction

The results of the two-sample MR analysis are presented as follows:inverse weighting analysis (β = 3.17/OR = 23.91, *p* = 0.02 < 0.05), MR-Egger (β = 2.25/OR = 9.51, *p* = 0.50), weighted median (β = 3.45/OR = 31.47, *p* = 0.05), simple model (β = 2.99/OR = 19.84, *p* = 0.37), and weighted model (β = 2.87/OR = 17.51, *p* = 0.31).

The inverse weighting analysis demonstrated a significant association between atorvastatin use and ED, as indicated by the *p*-value of 0.02 (<0.05). This finding was further supported by consistent OR (β) direction across all five statistical results.

These results suggest a potential causal relationship between atorvastatin and the development of ED, as indicated by the significant associations observed and the consistent direction of effect across different MR approaches.

**FIGURE 3:**
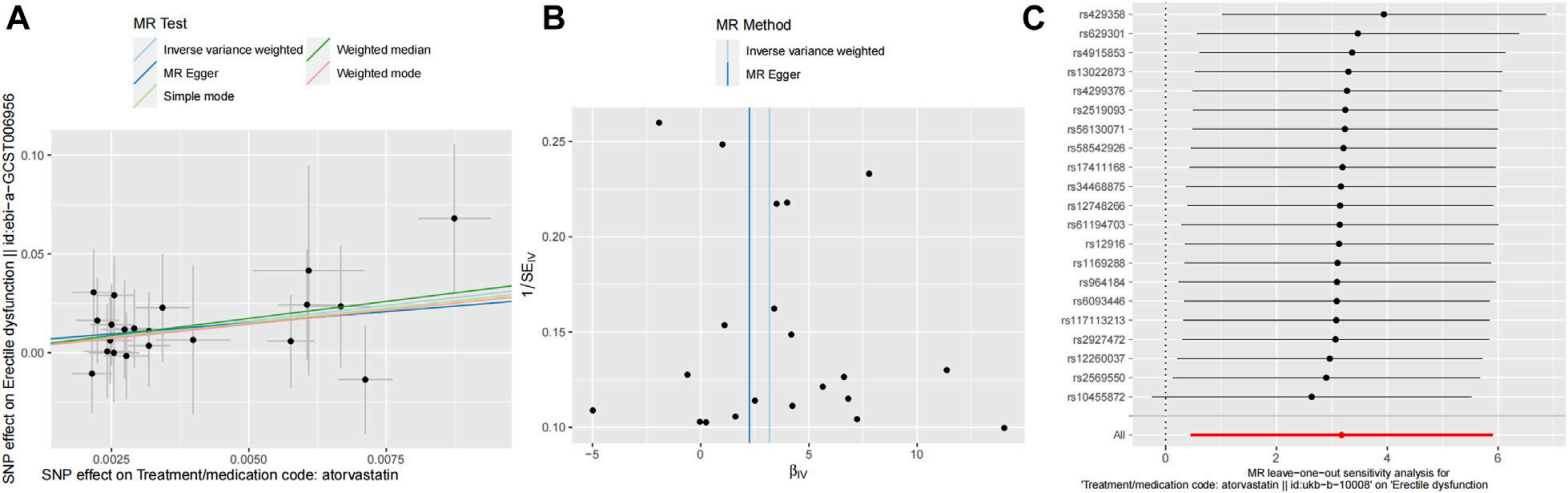
Mendelian randomized association between Atorvastatin and ED **(A)**: Scatter diagram, **(B)**: Funnel plot and **(C)**: Leave-one-out.

The funnel plot and leave-one-out sensitivity analyses, and scatter plots of MR analyses for Atorvastatin-ED shown in Figures 3A–C.

The Cochran’s Q test results were as follows: MR-Egger (*p* > 0.05) and inverse variance-weighted (*p* > 0.05). Our horizontal multi-effect analysis results were not statistically significant, with *p* > 0.05. However, these results exhibited good stability (Table 5).

**TABLE 5:** Pleiotropy test of Mendelian randomization results (Atorvastatin-ED).

Heterogene-test						Pleiotropy-test		
MR Egger			IVW			MR Egger		
Q	Q_df_	Q_*p*val_	Q	Q_df_	Q_*p*val_	Intercept	SE	*P* val
7.477130	19	0.9912027	7.573885	20	0.9943325	0.003789579	0.01218301	0.7591461

#### 3.2.2 LDL-C-erectile dysfunction

The two-sample MR analysis yielded the following results: inverse variance-weighted (β = 0.05 [OR = 1.05], *p* = 0.25), MR Egger (β = 0.04 [OR = 1.04], *p* = 0.50), weighted median (β = 0.11 [OR = 1.11], *p* = 0.10), simple model (β = 0.16 [OR = 1.18], *p* = 0.20), and weighted model (β = 0.10 [OR = 1.11], *p* = 0.14).

The inverse variance-weighted analysis results with *p* > 0.05 suggest that there is no significant causal relationship between LDL and ED. To assess the stability of the results, a sensitivity analysis was performed. The Cochran’s Q test results for MR-Egger (*p* > 0.05) and inverse variance-weighted (*p* > 0.05) were not significant, indicating good stability (Table 6).

**TABLE 6:** Pleiotropy test of Mendelian randomization results (LDL-ED).

Heterogene-test						Pleiotropy-test		
MR Egger			IVW			MR Egger		
Q	Q_df_	Q_*p*val_	Q	Q_df_	Q_*p*val_	Intercept	SE	*P* val
72.85954	77	0.6124935	72.88735	78	0.6424205	0.0006687527	0.004010455	0.8680021

**FIGURE 4:**
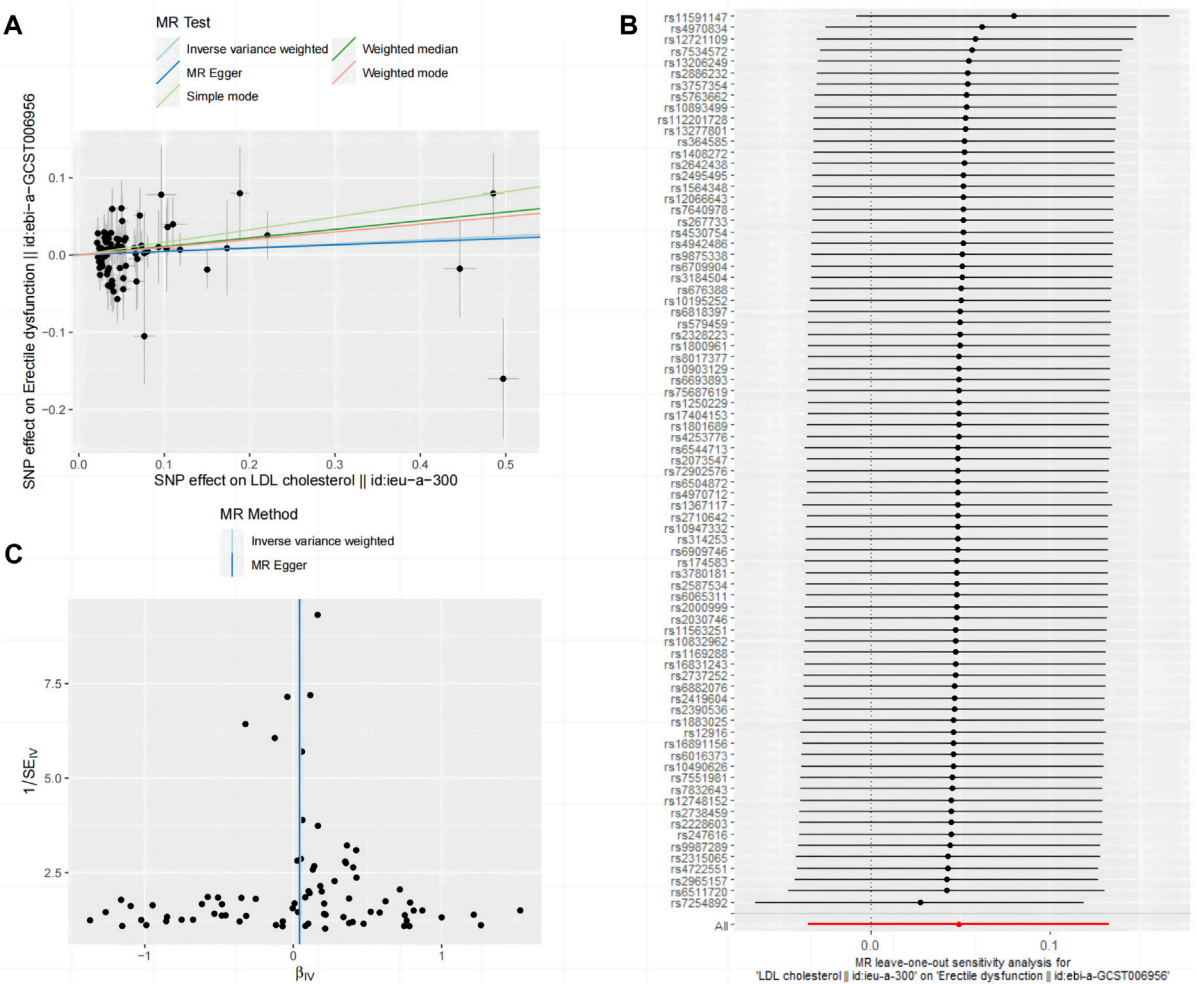
Mendelian randomized association between LDL and ED **(A)**: Scatter diagram, **(B)**: Leave-one-out and **(C)**: Funnel plot.

Figures 4A–C depict the funnel plot and leave-one-out sensitivity analyses, and scatter plots of the MR analyses conducted for the association between LDL and ED.

## 4 Discussion

Atorvastatin is one of the most widely used lipid-lowering drugs. Jabbari et al. found that atorvastatin can improve the prognosis of heart disease, by establishing a model-based cost-effectiveness analysis (Jabbari et al., 2020). The key pharmacological action of atorvastatin is the inhibition of rate-limiting enzymes in the mevalonate pathway (Graaf et al., 2004). This pathway is a key step in the synthesis of various compounds, including cholesterol and many nonsteroidal products. Jiang et al. established a mouse model of chronic subdural haemorrhage and found that atorvastatin accelerates the absorption of Chronic Subdural Hematoma by inducing an anti-inflammatory response and increasing Tregs in both the peripheral circulation and the brain (Quan et al., 2019). They found that a combination treatment using atorvastatin and the multikinase-targeting anticancer drug sorafenib synergistically inhibited proliferation and tumour sphere formation Gastric Cancer Stem Cells (GCSCs), in addition, GCSCs was therapeutically eliminated. This indicated that atorvastatin effectively inhibits formation of gastric cancer (Choi et al., 2022). Atorvastatin has a wide range of clinical effects; therefore, exploring its associated AEs is of practical significance.

Studies have found a potential association between atorvastatin and an increased risk of ED (Nurkalem et al., 2014). For example, a Finnish cohort study involving statin users showed a slightly elevated risk of initiating ED treatment after radical prostatectomy (Joentausta et al., 2023). Additionally, Solomon et al. discovered that patients with severe endothelial dysfunction were more likely to develop ED following statin therapy (Solomon et al., 2006). These findings suggest that ED may be an adverse reaction to atorvastatin.

Pharmacovigilance Analysis failed to verify a causal relationship between atorvastatin and ADR, as the disproportionate analysis only provided an estimate of signal strength and could neither quantify the risk nor establish causation (Zhao et al., 2023).

MR analysis employs genetic variations as IVs to evaluate the causal association between the exposure factor under study and the outcome of interest. This method has many unique advantages, such as avoiding confounding factors and high reliability (Larsson et al., 2023). Therefore, we combined the two methods, merging the ability of pharmacovigilance disproportion analysis to identify AEs with the advantage of MR for exploring causality. This provides more robust evidence for clinical decision-making. Through four pharmacovigilance analysis methods, this study found that ED (ROR:3.707078559,95%CI(3.332503494.123756054), (PRR:3.702969038,χ2 (669.2853829)), (IC:1.870490139,IC025(1.702813857), (EBGM:3.656567867,EBGM05 (3.28709656)) were effective ADRs, and it was the PT with the highest frequency. Demographic findings showed that adults aged 45–60 years were more likely to develop ED after taking atorvastatin. These people are also high-risk patients for ED in general (Corona et al., 2023).

The most common timeframe for the occurrence of AEs is within 0–30 days after administration of atorvastatin. Therefore, it is crucial to closely monitor patients’ sexual function during the first month of treatment. The majority of reports regarding AEs came from patients, followed by doctors. This observation may indicate that some physicians tend to overlook changes in patients’ sexual function during their clinical practice. Additionally, our MR investigation indicates a possible association between atorvastatin and ED. These results align with the outcomes derived from our pharmacovigilance monitoring analysis. Therefore, combining these two approaches can effectively explore the adverse effects of most drugs, providing more comprehensive insights into their safety profiles.

ED is a significant vascular condition linked closely with risk factors like hyperlipidemia, metabolic syndrome, diabetes, inflammation, hypertension, and vascular endothelial function (Balta and Mikhailidis, 2019). Research by Ma et al. indicates that elevated LDL levels can increase the incidence of ED in youthful males (Ma et al., 2021). Currently, nitric oxide (NO) synthesis and release play a crucial role in erectile function, being considered the primary factor in the relaxation of penile blood vessels and the corpus cavernosum (Andersson, 2011). Statin drugs induce and regulate endothelial nitric oxide synthase to increase NO production *in vitro* (Tousoulis et al., 2014). Research by Roberto suggests that PCSK9 inhibitors significantly improve lipid levels in male patients with familial hypercholesterolemia, thereby enhancing erectile function (Scicali et al., 2020). A double-blind randomized controlled trial found that treatment with simvastatin for 6 months improved erectile dysfunction (Trivedi et al., 2014). A prospective study compared the effects of rosuvastatin and atorvastatin on erectile dysfunction. They observed that atorvastatin increased the risk of erectile dysfunction (Nurkalem et al., 2014). These results suggest that different statin types may have different effects on erectile dysfunction. However, Akdeniz et al. observed a significant reduction in the number of spermatogonia and spermatocytes in male Sprague-Dawley rats following 12 weeks of atorvastatin intervention compared to the comparison group (Akdeniz et al., 2021). In their animal experiments, Bolat et al. found that atorvastatin can rapidly decrease intracavernosal pressure under 10 V stimulation in rats, lower testosterone levels, and impact sexual function (Bolat et al., 2019). In their study, Baspınar et al. found that patients on statin therapy might observe a rise in NO levels; however, a decrease in NO levels could occur when their LDL-C levels reach 100 mg/dL. This decline in NO levels at lower LDL-C concentrations could potentially worsen the incidence of ED (Baspınar et al., 2016). This may explain why ED occurs with atorvastatin rather than other types of lipid-lowering drugs.

Atorvastatin may also cause ED because of its effect on testosterone, with studies suggesting that atorvastatin may negatively affect erectile function by interfering with testosterone synthesis and metabolism and reducing testosterone levels (Bolat et al., 2019). This impact could be linked to the hindrance of the cholesterol synthesis pathway, given that cholesterol serves as a vital precursor for testosterone synthesis (Dobs et al., 2000).

Nevertheless, it is crucial to emphasize the need for additional research to confirm these mechanisms and investigate other potential variables that could impact the association between atorvastatin and ED. Factors such as individual variations, dosage, and duration of atorvastatin use should also be considered in understanding this association. Further clinical trials and lab investigations will enhance our grasp of the intricate connection between atorvastatin and ED.

Atorvastatin is often used in combination with cardiovascular drugs as a lipidlowering agent. CVD and ED share common risk factors and pathophysiological associations, such as endothelial dysfunction and inflammation (Terentes-Printzios et al., 2021). Current studies suggest a complex interaction between ED and CVD drugs. Studies have shown that cardiovascular medications such as beta-blockers can cause ED (Silvestri et al., 2023). Therefore, a simple pharmacovigilance analysis may be inaccurate. This demonstrates the limitations of pharmacovigilance analysis of real-world data. Therefore, based on the results of pharmacovigilance analysis, we introduced Mendelian randomization to further clarify the causal relationship. To further provide evidence for the relationship between atorvastatin and ED.

The efficacy and safety data for atorvastatin were initially derived from preclinical trials. Because of the relatively small sample sizes used, however, Enrolled clinical trials may not completely mirror the real-world effects of medications on individuals, especially concerning safety aspects (Zhou et al., 2023).

FAERS is dynamically updated and open to the public as a post-marketing drug safety monitoring and reporting system (Moore et al., 2020). Continuous attention to adverse drug events is helpful for evaluating the safety of drugs and making the best choice for patients during clinical decision-making. Using FAERS for pharmacovigilance analysis has the advantages of being multi-centre, providing large amounts of data, and containing many samples. However, our study has some limitations. FAERS is an open database of self-reporting systems; hence the data quality is not standardised, owing to simple differences between uploaders. This may lead to deviations in the results. On the side, confounding factors, Factors like medication dosage, treatment duration, existing health conditions, drug interactions, and specific disease types pose challenges in terms of control.

## 5 Conclusion

Through thorough analyses integrating pharmacovigilance and MR, our results indicate a potential causal link, suggesting that using atorvastatin is linked to a heightened risk of ED. These findings underscore the significance of taking into account possible side effects, like ED, when prescribing atorvastatin for the prevention of cardiovascular disease. The combination of pharmacovigilance monitoring and MR holds great promise in identifying previously unknown or underreported ADRs. It offers a systematic and evidence-based approach to evaluate drug safety and contributes to the ongoing efforts in improving patient care and drug regulation.

## Data Availability

Publicly available datasets were analyzed in this study. This data can be found here: https://gwas.mrcieu.ac.uk/ and https://www.fda.gov/drugs/drug-approvals-and-databases/fda-adverse-event-reporting-system-faers.
